# Effect of MTA and CEM on Mineralization-Associated Gene Expression in Stem Cells Derived from Apical Papilla

**DOI:** 10.22037/iej.v13i1.17860

**Published:** 2018

**Authors:** Niusha Hajizadeh, Zahra Sadat Madani, Ebrahim Zabihi, Moniyreh Golpour, Amir Zahedpasha, Mousa Mohammadnia

**Affiliations:** a *Department of Endodontics, School of Dentistry, Babol University of Medical Sciences, Babol, Iran;*; b *Dental Materials Research Center, School of Dentistry, Babol University of Medical Sciences, Babol, Iran; *; c *Cellular and Molecular Biology Research Center, Health Research Institute, Babol University of Medical Sciences, Babol, Iran; *; d *Molecular and Cell Biology Research Center, Student Research Committee, Medical School, Mazandaran University of Medical Sciences, Sari, Iran; *; e *Department of Oral and Maxillofacial Surgery, School of Dentistry, Babol University of Medical Sciences, Babol, Iran; *; f *Department of Immunology, School of Medicine Babol University of Medical Sciences, Babol, Iran *

**Keywords:** Biomaterials, Calcium-Enriched Mixture (CEM Cement), Mineral Trioxide Aggregate, Relative Gene Expression, Retrograde Root Filling Materials, Stem Cells from Apical Papilla

## Abstract

**Introduction:**

This study assessed the effect of mineral trioxide aggregate (MTA) and calcium-enriched mixture (CEM) cement on odontogenic differentiation and mineralization of stem cells.

**Methods and Materials:**

After confirmation of stemness and homogeneity of stem cells derived from apical papilla (SCAPs) using flow cytometry, the cells were exposed for 3 weeks to either osteogenic medium (OS) or CEM extract+OS (CEM+OS) or MTA extract in OS (MTA+OS) or DMEM based regular culture media (negative control). Relative expression of alkaline phosphatase (ALP), dentine sialophosphoprotein (DSPP), osteocalcin (OSC), and osterix (SP7) were measured at days 14 and 21 using RT-qPCR method. At the same time points Alizarin Red staining method was used to assess mineralization potential of SCAPS. Gene expression changes analysis were made automatically using REST® software and a *P*<0.05 was considered significant.

**Results:**

After 2 weeks of exposure, expression of all genes were between 3 and 52 times the expression of GADPH (all were upregulated except SP7 in the control,* P*<0.05). After 3 weeks, relative expressions of the genes: ALP, SP7, DSPP, and OSC were respectively 275.9, 528.3, 98.4, and 603.7 times the expression of GADPH in the control group (OS). These were respectively 17.405, 29.2, 11.8, and 6.5 in CEM+OS group, and 163.8, 119.7, 102.5, and 723.9 in MTA+OS group. All of these were confirmed as upregulated (*P*<0.05) except for ALP and OSC of DM+CEM group. After 2 weeks, alizarin red staining showed similar mineralized nodules in OS, MTA+OS, and CEM+OS. In third week, larger nodules were seen in MTA+OS and OS, but not in CEM+OS.

**Conclusion:**

After 2 weeks, gene expressions were almost comparable in OS, CEM+OS, and MTA+OS. After 3 weeks, OS and MTA+OS upregulated genes much greater than in 2nd week. However, upregulation in CEM+OS might not increase in 3rd week compared to those in 2nd week.

## Introduction

Maintaining the pulp vitality is crucial for apex closure of immature permanent teeth. Trauma or pulp infection can halt the development of an immature root and leave the apex open. Conventional treatments for such cases were to remove the pulp, use calcium hydroxide in the canal every three months to induce apexification, and obturation of the incomplete but apexified canal. Regenerative endodontic treatment is a new successful alternative for completion of the root development and apex closure (particularly when the pulp damage is due to trauma) through regaining the pulp vitality by differentiation of stem cells and continuation of mineralization [1-5]. It is advantageous over the conventional method, as unlike apexification, which needs numerous sessions of calcium hydroxide administration, it needs a short-term treatment period; also unlike apexification, this method induces physiological development of the root structure and increases in root length and thickness [5-9]. Root-end filling materials are a major part of these regenerative and open-apex treatments [1-3]. These materials are also used when the conventional (non-surgical) endodontic treatment fails and alternative root-end surgical approaches become necessary. They can affect the treatment success and thus they should have various proper characteristics such as dimensional stability, resistance to resorption and moisture, and adherence to cavity walls. Moreover, due to being in direct contact to live tissues, they need to be biocompatible and non-toxic in order to promote healing [1, 10-12]. Also as a part of the newly introduced “regenerative treatments”, they should be able to induce differentiation of stem cells and motivate hard tissue formation [13, 14].

Several root-end filling materials have been marketed to replace the amalgam, which has drawbacks such as dimensional changes, corrosion, unsightly tattoos, and low biocompatibility [2, 12, 15]. These include mineral trioxide aggregate (MTA) and a recently introduced cement which is rich in calcium (calcium-enriched mixture or CEM cement) [2, 3]. MTA is a common root end filling material that despite proper biocompatibility has drawbacks such as a high price, long setting times, coronal discoloration, and not-easy handling [3, 10-12, 15-21]. To overcome some of these drawbacks, CEM cement was introduced; it has proper sealing abilities comparable or better than that of MTA [22, 23]. Unlike MTA, this cement can induce the formation of hydroxyapatite crystals similar to the standard crystals over its surface when stored in or close to normal saline [24]. Therefore, it might be better for bone morphogenesis or sealing the root apex which may be due to release of calcium and phosphor ions [25]. In addition, CEM can produce more hydroxyapatite crystals in phosphate buffered saline which is similar to the interstitial fluid [26]. Furthermore, it has important physicochemical and clinical properties such as shorter setting times, less coronal discoloration, better fluidity, and a smaller film thickness [22, 24].

Hard tissue formation in the vicinity of the root end filling materials requires the differentiation of pulp stem cells and activation of mineralization process [27]. Stem cells of apical papilla (SCAP) of the teeth play an important role in bone and dental tissue regeneration during treatments such as apexogenesis and regeneration of immature teeth. Odontogenic or osteoblastic differentiation of these cells is influenced by environment factors such as IGF-1, resin monomers, MTA and other chemicals that can induce mineralization [13, 14]. Studies on gene expression of stem cells under the influence of CEM are limited to few short term studies (mostly 3 days, with few 1- or 2-week periods) [28, 29]. Moreover the effects of MTA or other root-end filling materials on many osteogenesis-related genes are not evaluated before. Hence, this study was conducted to assess the effect of MTA, CEM cement, and Dulbecco’s modified Eagle medium (DMEM) supplemented with osteogenic materials as positive control [30] on the differentiation and mineralization of SCAPs using quantitative real-time polymerase chain reaction (QPCR) of 4 genes and alizarin red methods during 3 weeks.

## Materials and Methods

***Stem cell culture***

The apical papilla was collected from an impacted and immature third molar of a 16-year-old boy, extracted aseptically for reasons other than this research, and placed immediately in sterile Hank's solution (with Pen/Strep). The tooth was obtained with the consent of the patient and in accordance with the Helsinki declaration. Ethics of the study were approved by the ethics committee of Babol University of Medical ‎Sciences, Babol, Iran. The tooth was cleansed off debris and blood cells using ethanol, followed by PBS and HPSS. 

The root apical papilla was gently withdrawn from the apex. After gently slicing the papilla tissue and adding 3 mg/mL of type I collagenase to it, it was incubated at 37^°^C for 3 h in order to separate the cells. Once the whole tissue was disintegrated in the collagenase solution, the culture was sieved through a 70 µm cell strainer (BD, USA) in order to create a homogenous cell suspension. The suspension was centrifuged at 250 g and 4^°^C for 7 min. The collected cells were cultured in DMEM-High Glc (Gibco, USA)+FBS 10% (Gibco, USA)+PenStrep %1 (Atocell, Austria), and incubated (37^°^C, 5% CO_2_, and 95% humidity). Every 2 days, the culture medium (10 mL) was refreshed after washing the cells with PBS in a 75-cm^2^ flask. This was done until the second passage [31]. 

***Stem cell characterization***

SCAPs gross morphology was evaluated using an inverted light microscope (Olympus, Tokyo, Japan). Expression of cell surface antigens CD45 (cat No.17-0459), CD34 (cat No.12-0349), CD73 (cat No.46-0739), CD90 (cat No.11-0909), and CD105 (cat No.17-105) in stem cells of apical papilla was assessed using flow cytometry (FACS Calibur Flow Cytometer, Becton Dickinson, CA, USA) and eBioscience kits (eBioscience, USA) .

**Figure 1:**
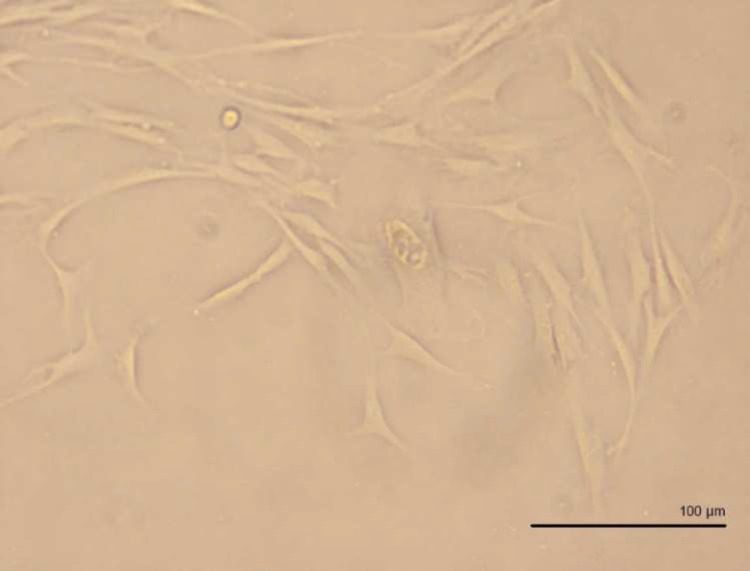
Shiny and live spindle-like stem cells derived from human apical papilla (SCAP) micrograph taken by inverted phase contrast light microscope (400×)

***Preparation of material extracts and cell exposure***

One g from ProRoot MTA (Denstply Tulsa Dental, Tulsa, OK, USA) and 1 g from CEM cement (BioniqueDent, Tehran, Iran) were prepared in sterile condition according to the manufacturer’s instructions, in a 24-well cell culture plate. Then they were left to be set according to the instructions of the manufacturers overnight in a humid CO_2_ incubator. Afterwards, 1 mL of plain culture medium (DMEM-High Glc) (Gibco Laboratories, Grand Is., NY, USA) was added to each well. The plate was incubated at 37 C, 5% CO_2_, and 95% humidity for 72 h. Then the supernatant medium was gently suctioned to be used for stem cell differentiation medium preparation by adding 10% FBS, 1% Pen/Strep, and supplementing with osteogenic reagents (50 mg/mL ascorbic 2-phosphat, 10 nM dexamethasone, and 3 mM β glycerol phosphate). The above 72 h extract of the two cements (supplemented with osteogenic reagents),‎were exposed to the cells (2 mL/well) in a 12-well plate for 72 h then repeated every 72 h up to 3 weeks. The experiments were carried in triplicates. 

***Osteogenic and odontoblastic differentiation of stem cells***

Nine wells of a 12-well plate were used to culture the SCAPs of the 2^nd^ subculture. In each well, about 10^4^ cells were seeded and 2 mL of DMEM-high Glc supplemented with 10% FBS, 1% PenStrep was added to each well. Six of the 9 wells, were used for the test cements extracts in osteogenic medium, and 3 were used as positive control (osteogenic medium alone) (OS). As the negative control DMEM-High Glc medium supplemented with 10% FBS and 1% PenStrep was added to 3 remaining wells. Each plate was incubated for 3 weeks in a humid CO_2_ incubator and the freshly prepared media with/without extracts were replaced every 72 h. 

***Alizarin red assessment of mineralization***

In the day 14^th^ and 21^st^, stem cells from each of 9 wells were stained with alizarin red. Then they were evaluated under light microscopy (Olympus, Tokyo, Japan) [30]. 

***Real-time PCR assessment of osteogenic and odontoblastic differentiation***

In the 14^th^ and 21^st^ days, stem cells from each well were sampled and subjected to RT-qPCR assessment. Cell differentiation was assessed using designed primers for cDNA of the following genes: alkaline phosphatase (ALP), dentine sialophosphoprotein (DSPP), osteocalcin (OSC), osterix (SP7), and glyceraldehyde-3-phosphate dehydrogenase (GAPDH, as reference gene) [31]. Briefly, the RNA was extracted(TotalRNA Extraction Mini Kit, FavorGen, Taiwan). Its quality was assessed with electrophoresis on agarose gel and its pureness and concentration were assessed using a spectrophotometer (NanoDrop, Thermo, USA) at 260/280 nm wavelengths. After ensuring a proper quality of extracted RNA, corresponding cDNAs were replicated (CinnaGen First Strand cDNA Synthesis Kit). Expression of the above mentioned genes was assessed in 20 µL reaction mixture (cDNA product 1 µL+predesigned primers 0.5 µL+Milli-Q water 8.5 µL+Master Mix 10 µL) using real-time PCR method (ABI 7300, Super SYBR Green qPCR Master Mix 2x, FavorGen, Taiwan) for one cycle of 30 sec at 95 C followed by 40 cycles of 90 sec each (30 seconds at 95^°^C followed by 30 sec at 60 C and 30 sec at 72^°^C per cycle). A microarray designer software (AllelD 6.0, Premium Biosoft, Palo Alto, CA, USA) was used to design the sequence of the used primers (Table 1). Comparative CT method (2^ΔΔCt^) was used for analyzing the obtained qPCR data. The relative expression of each gene to GAPDH was compared in different groups at two time points

**Table 1:** Primer sequences used in gene expression analyses

**Gene**	**Sequence (5' -> 3')**	**Product size (bp)**
**ALP F**	ACAAGCACTCCCACTTCATCTGGA	126
**ALP R**	TCACGTTGTTCCTGTTCAGCTCGT	
**DSPP F**	CAACCATAGAGAAAGCAAACGCG	120
**DSPP R**	TTTCTGTTGCCACTGCTGGGAC	
**OSC F**	GGCGCTACCTGTATCAATGG	110
**OSC R**	GTGGTCAGCCAACTCGTCA	
**SP7 F**	TGCTTGAGGAGGAAGTTCAC	148
**SP7 R**	AGGTCACTGCCCACAGAGTA	
**GAPDH F**	GGTGGTCTCCTCTGACTTCAACA	127
**GAPDH R**	GTTGCTGTAGCCAAATTCGTTGT	

***F*** *, forward;* ***R*** *, reverse*

**Figure 2:**
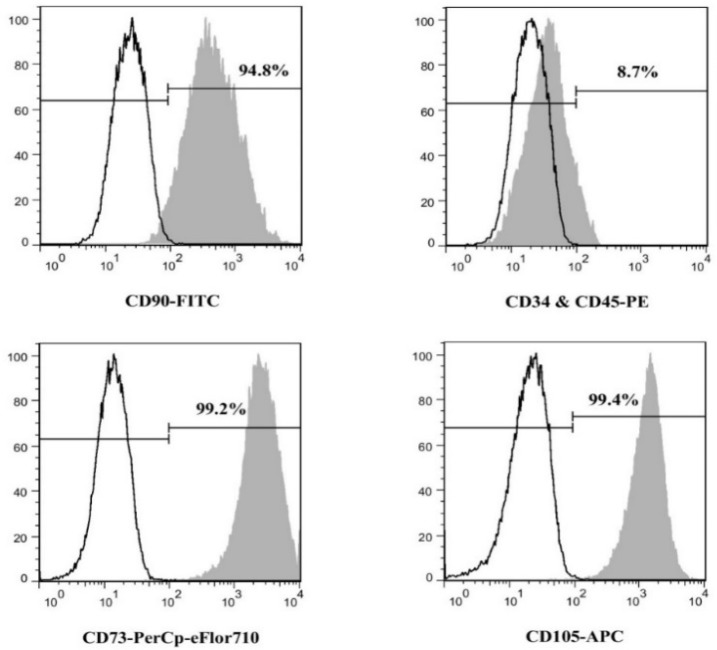
Results of flow cytometry, showing presence of mesenchymal stemness markers (CD90, CD73, and CD105) while lacking hematopoietic markers (CD34 and CD45) surface antigens

***Statistical analysis***

The mean of expression fold change for different osteogenic genes, measured in triplicate, have been calculated and Statistical analysis consequent top air wise fixed reallocation randomization test *via* a complex Taylor algorithm using Relative Expression Software Tool (REST, version 2009, QIAGEN, Germany) were performed. One-Way ANOVA and unpaired *t*-test were performed using SPSS version 18.0 (SPSS, Chicago, IL, USA). Level of significance was predetermined at 0.05. 

## Results

***Stem cell characteristics***

All images of light microscopy showed spindle-form stem cells morphology (Figure 1). Flow cytometry showed expressions of CD73, CD90, and CD105 and lack of CD45 and CD34 expressions (Figure 2).

***Alizarin red***

**Two weeks**

**Figure 3:**
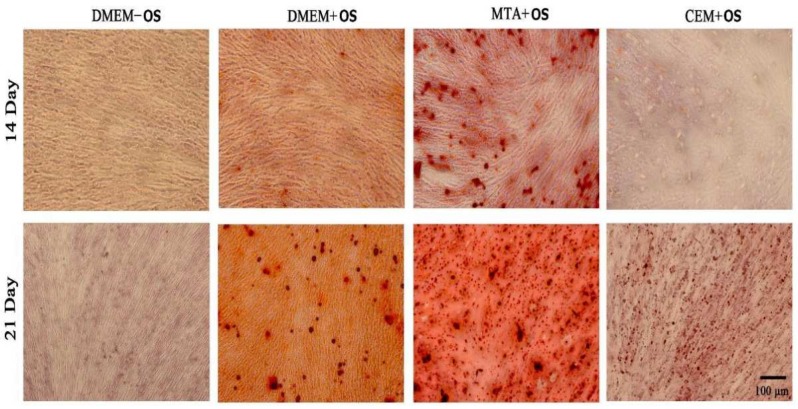
Micographs (10× magnification) of osteogenic differentiation of stem cells derived from apical papilla (SCAP) stained by alizarin red 14 & 21 days after exposure to: culture medium without osteogenic reagents (DMEM-OS), culture medium supplemented with osteogenic reagents (DMEM+OS), MTA extract in osteogenic medium (MTA+OS), and CEM extract in osteogenic medium (CEM+OS) (DMEM+OS)

Microscopic evaluation showed more mineralization with MTA+OS (MTA within osteogenic medium), and less mineralization with CEM+OS compared to the positive control (OS) group, while could be seen with showed mineralization compared to MTA at this time point (Figure 3). 

**Three weeks**

MTA+OS and positive control groups showed different sizes of stained nodules ranging from smaller to larger nodules. However, CEM+OS showed only small nodules (Figure 3).

***Real-time PCR***

**Two weeks**

After 2 weeks, all the evaluated 4 genes were significantly up-regulated in all 3 groups, except SP7 in control OS alone group (Figure 3). 

**Three weeks**

After 3 weeks, all genes except ALP and OSC of CEM+OS group were up regulated (Figure 3).

## Discussion

The findings of this study indicated that the differentiation medium alone or together with MTA or CEM extract could up regulate the expression of 4 evaluated genes crucial for bone or dentine formation. Actually the differentiation medium alone had the best results. The genes evaluated are responsible for osteogenesis and dentinogenesis. ALP plays an important role in mineralization of reparative dentin and is used for the detection of early osteogenic differentiation and bone turnover [32-35]. DSPP expression occurs once the collagenous predentin matrix is formed and is indicative of mature osteoblast and associated with dentinogenesis [35-39]. Osteocalcin is a major noncollagenous protein of dentin and bone which is secreted only by cells of mineralizing capacity (cementoblasts, odontoblasts, osteoblasts) and plays a regulatory role in the mineralization of hard tissue [35, 40-44]. The results of the current study regarding MTA were in line with earlier research showing increases in odontogenic, cementogenesis, and dentinogenesis activity [17, 28, 45-49]. 

However, unlike other studies [17, 45, 46, 48, 49], CEM did not show an overall increase in the expression of these genes, which needs future studies.

A noteworthy finding was that after 3 weeks, the expressions in the CEM group were much lower than gene expressions observed in the differentiation medium alone (control group) or that with MTA extract. This might imply some adverse effect attributed to CEM that could reduce gene expression as a result of interfering with normal cell differentiation. CEM has shown to have proper effects in pulpotomy in long term [50, 51]. It has also been shown biocompatible within 1 day [30], 3 days [29, 52, 53], 1 week [54, 55], or 2 weeks [28]. In one study, it was shown that bone response to CEM cement implanted in rats might be similar to the response to implanted MTA one week post-surgery [48]. Ahangari *et al*. [30] compared MTA with CEM cement regarding the osteoblastic differentiation of bone marrow-derived mesenchymal stem cells by means of comparing the expression of alkaline phosphatase enzyme as well as type I collagen and osteocalcin. They reported no significant difference between the expression of type I collagen but a significant superiority of osteocalcin gene in the CEM group, indicating a higher potential of CEM compared to MTA in induction of mineralization [30]. 

According to the present study, although CEM can induce stem cell differentiation within 2 weeks, it might not increase gene expression more, in the third week. Since our study lacked methods capable of directly assessing cytotoxicity (like MTT assay), future studies are required to evaluate long-term cytotoxicity of CEM on SCAPs. On the other hand, MTA and positive control showed increasing upregulation of genes in both the second and third weeks. These results were confirmed by alizarin red staining that showed a prominent extent of mineralized matrices in MTA and control groups but not in CEM group, similar to another study [28]. The down-regulation of gene expression in CEM-exposed cultures to the existence of a negative-feedback loop in mRNA expression after initiation of mineralization; however, this needs future studies.

Biocompatibility of MTA is documented [3, 10-12, 15, 20, 21], and even it has been considered by some authors as the gold standard for assessment of biocompatibility of other root-end materials [1, 11, 56]. However, findings regarding its biocompatibility are actually quite controversial ranging from very good to extremely poor results [1, 3, 10-12, 15, 20, 21, 56-58]. It might stimulate upregulation of collagen and prostaglandin gene expression as well as induction of cell growth and production of more mineralized matrix, probably due to releasing high levels of calcium and silicate ions which are its main components [1, 12, 15, 18, 59]. It also might reduce inflammation and support cementum deposition [12]. However, the findings of contrasting studies denote that MTA might have suppressing effects on cell growth [57, 59], possibly due to discharge of cytotoxic ions such as aluminum and bismuth [59]. MTA is moisture-insensitive, can induce proliferation of fibroblasts and mineralization of osteoblasts, and seems to be biocompatible [58-60]. Freshly setting MTA may increase the pH of the culture from 7.2-10.2 after mixing, to 10.2-12.5 after 24 h; this can inhibit gene expression as well as causing cell lysis and medium protein denature in short term [2, 3, 58]. 

This study was limited by some factors. Unlike other studies, we did not compare gene expressions in different groups, because the groups were small (due to high accuracy of PCR), and not appropriate for conducting statistical tests. Again unlike previous studies which implied gene upregulation by comparing with control, we directly evaluated the upregulation of genes by comparing them with a combination of reference gene and negative control using a complicated statistical algorithm. We also provided 95% confidence intervals which are more flexible than *P*-values for comparing groups especially when the groups are of unknown sizes (in previous studies). Most previous studies have not reported the sample sizes, and we doubt PCR groups are greater than 3 or 4 specimens, which is not large enough for statistical comparisons of sufficient power.

**Figure 4:**
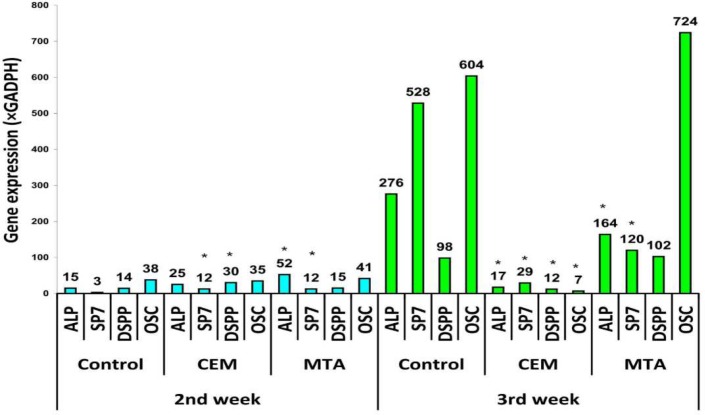
Expression fold change in different osteogenic genes relative to gene expression of GADPH **(**as negative control) acquired from ΔΔCt analysis procedure in qPCR test, * *P*<0.05 (t-test comparison with average of positive control)

## Conclusion

The MTA extract in osteogenic medium up regulated the expression of the evaluated genes, especially osteocalcin. Although CEM extract showed elevated expression of genes compared to negative control in the second week, the gene expression was not enhanced further in the third week. The osteogenic medium alone showed an up regulating effect higher than that of MTA and CEM on all the evaluated genes except for osteocalcin (which was slightly lower than that of MTA). The SCAP mineralization of induced by MTA extract and osteogenic medium but not CEM was remarkable.

## References

[1] Al-Sa'eed OR, Al-Hiyasat AS, Darmani H (2008). The effects of six root-end filling materials and their leachable components on cell viability. J Endod.

[2] Chong BS, Pitt Ford TR (2005). Root-end filling materials: rationale and tissue response. Endodontic Topics.

[3] Haglund R, He J, Jarvis J, Safavi KE, Spangberg LS, Zhu Q (2003). Effects of root-end filling materials on fibroblasts and macrophages in vitro. Oral Surg Oral Med Oral Pathol Oral Radiol Endod.

[4] Lee BN, Lee KN, Koh JT, Min KS, Chang HS, Hwang IN, Hwang YC, Oh WM (2014). Effects of 3 endodontic bioactive cements on osteogenic differentiation in mesenchymal stem cells. J Endod.

[5] Lee BN, Moon JW, Chang HS, Hwang IN, Oh WM, Hwang YC (2015). A review of the regenerative endodontic treatment procedure. Restor Dent Endod.

[6] Banchs F, Trope M (2004). Revascularization of immature permanent teeth with apical periodontitis: new treatment protocol?. J Endod.

[7] Goyal L (2014). Clinical effectiveness of combining platelet rich fibrin with alloplastic bone substitute for the management of combined endodontic periodontal lesion. Restor Dent Endod.

[8] Hotwani K, Sharma K (2014). Platelet rich fibrin-a novel acumen into regenerative endodontic therapy. Restor Dent Endod.

[9] Shah N, Logani A, Bhaskar U, Aggarwal V (2008). Efficacy of revascularization to induce apexification/apexogensis in infected, nonvital, immature teeth: a pilot clinical study. J Endod.

[10] Al-Qathami H, Balto H, Al-Nazha S, Siddiqui Y (2004). Effect of root perforation repair materials on morphology and attachment behavior of human PDL fibroblasts in vitro. Saudi Dental Journal.

[11] Balto HA (2004). Attachment and morphological behavior of human periodontal ligament fibroblasts to mineral trioxide aggregate: a scanning electron microscope study. J Endod.

[12] Bonson S, Jeansonne BG, Lallier TE (2004). Root-end filling materials alter fibroblast differentiation. J Dent Res.

[13] Bakopoulou A, Leyhausen G, Volk J, Tsiftsoglou A, Garefis P, Koidis P, Geurtsen W (2011). Comparative analysis of in vitro osteo/odontogenic differentiation potential of human dental pulp stem cells (DPSCs) and stem cells from the apical papilla (SCAP). Arch Oral Biol.

[14] Srisuwan T, Tilkorn DJ, Wilson JL, Morrison WA, Messer HM, Thompson EW, Abberton KM (2000). Molecular aspects of tissue engineering in the dental field. Periodontol.

[15] Pistorius A, Willershausen B, Briseno Marroquin B (2003). Effect of apical root-end filling materials on gingival fibroblasts. Int Endod J.

[16] Chng HK, Islam I, Yap AU, Tong YW, Koh ET (2005). Properties of a new root-end filling material. J Endod.

[17] Tabarsi B, Parirokh M, Eghbal MJ, Haghdoost AA, Torabzadeh H, Asgary S (2010). A comparative study of dental pulp response to several pulpotomy agents. Int Endod J.

[18] Tani-Ishii N, Hamada N, Watanabe K, Tujimoto Y, Teranaka T, Umemoto T (2007). Expression of bone extracellular matrix proteins on osteoblast cells in the presence of mineral trioxide. J Endod.

[19] Torabinejad M, Parirokh M (2010). Mineral trioxide aggregate: a comprehensive literature review--part II: leakage and biocompatibility investigations. J Endod.

[20] Balto H, Al-Nazhan S (2003). Attachment of human periodontal ligament fibroblasts to 3 different root-end filling materials: Scanning electron microscope observation. Oral Surg Oral Med Oral Pathol Oral Radiol Endod.

[21] Pelliccioni GA, Ciapetti G, Cenni E, Granchi D, Nanni M, Pagani S, Giunti A (2004). Evaluation of osteoblast-like cell response to Proroot MTA (mineral trioxide aggregate) cement. J Mater Sci Mater Med.

[22] Asgary S, Eghbal MJ, Parirokh M (2008). Sealing ability of a novel endodontic cement as a root-end filling material. J Biomed Mater Res A.

[23] Yavari HR, Samiei M, Shahi S, Aghazadeh M, Jafari F, Abdolrahimi M, Asgary S (2012). Microleakage comparison of four dental materials as intra-orifice barriers in endodontically treated teeth. Iran Endod J.

[24] Asgary S, Eghbal MJ, Parirokh M, Ghoddusi J, Kheirieh S, Brink F (2009). Comparison of mineral trioxide aggregate's composition with Portland cements and a new endodontic cement. J Endod.

[25] Asgary S, Eghbal MJ, Parirokh M, Ghoddusi J (2009). Effect of two storage solutions on surface topography of two root-end fillings. Aust Endod J.

[26] Asgary S, Ahmadyar M (2013). Vital pulp therapy using calcium-enriched mixture: An evidence-based review. J Conserv Dent.

[27] Economides N, Pantelidou O, Kokkas A, Tziafas D (2003). Short-term periradicular tissue response to mineral trioxide aggregate (MTA) as root-end filling material. Int Endod J.

[28] Asgary S, Nazarian H, Khojasteh A, Shokouhinejad N (2014). Gene expression and cytokine release during odontogenic differentiation of human dental pulp stem cells induced by 2 endodontic biomaterials. J Endod.

[29] Ghasemi N, Rahimi S, Lotfi M, Solaimanirad J, Shahi S, Shafaie H, Salem Milani A, Shakuie S, Zand V, Abdolrahimi M (2014). Effect of Mineral Trioxide Aggregate, Calcium-Enriched Mixture Cement and Mineral Trioxide Aggregate with Disodium Hydrogen Phosphate on BMP-2 Production. Iran Endod J.

[30] Hengameh A, Reyhaneh D, Nima MM, Hamed H (2014). Effects of two bioactive materials on survival and osteoblastic differentiation of human mesenchymal stem cells. J Conserv Dent.

[31] Wang L, Yan M, Wang Y, Lei G, Yu Y, Zhao C, Tang Z, Zhang G, Tang C, Yu J, Liao H (2013). Proliferation and osteo/odontoblastic differentiation of stem cells from dental apical papilla in mineralization-inducing medium containing additional KH(2)PO(4). Cell Prolif.

[32] Chen CC, Shie MY, Ding SJ (2011). Human dental pulp cell responses to new calcium silicate-based endodontic materials. Int Endod J.

[33] Kulterer B, Friedl G, Jandrositz A, Sanchez-Cabo F, Prokesch A, Paar C, Scheideler M, Windhager R, Preisegger KH, Trajanoski Z (2007). Gene expression profiling of human mesenchymal stem cells derived from bone marrow during expansion and osteoblast differentiation. BMC Genomics.

[34] Larmas M (2008). Pre-odontoblasts, odontoblasts, or "odontocytes". J Dent Res.

[35] Rathinam E, Rajasekharan S, Chitturi RT, Martens L, De Coster P (2015). Gene Expression Profiling and Molecular Signaling of Dental Pulp Cells in Response to Tricalcium Silicate Cements: A Systematic Review. J Endod.

[36] Feng JQ, Luan X, Wallace J, Jing D, Ohshima T, Kulkarni AB, D'Souza RN, Kozak CA, MacDougall M (1998). Genomic organization, chromosomal mapping, and promoter analysis of the mouse dentin sialophosphoprotein (Dspp) gene, which codes for both dentin sialoprotein and dentin phosphoprotein. J Biol Chem.

[37] MacDougall M, Nydegger J, Gu TT, Simmons D, Luan X, Cavender A, D'Souza RN (1998). Developmental regulation of dentin sialophosphoprotein during ameloblast differentiation: a potential enamel matrix nucleator. Connect Tissue Res.

[38] Min KS, Lee SI, Lee Y, Kim EC (2009). Effect of radiopaque Portland cement on mineralization in human dental pulp cells. Oral Surg Oral Med Oral Pathol Oral Radiol Endod.

[39] Zhang S, Yang X, Fan M (2013). BioAggregate and iRoot BP Plus optimize the proliferation and mineralization ability of human dental pulp cells. Int Endod J.

[40] Khoshzaban A, Rakhshan V, Najafi F, Aghajanpour L, Hashemian SJ, Keshel SH, Watanabe I, Valanezhad A, Jafarzadeh Kashi TS (2017). Effect of sintering temperature rise from 870 to 920 degrees C on physicomechanical and biological quality of nano-hydroxyapatite: An explorative multi-phase experimental in vitro/vivo study. Mater Sci Eng C Mater Biol Appl.

[41] Saygin NE, Giannobile WV, Somerman MJ (2000). Molecular and cell biology of cementum. Periodontol.

[42] Sun H, Wu C, Dai K, Chang J, Tang T (2006). Proliferation and osteoblastic differentiation of human bone marrow-derived stromal cells on akermanite-bioactive ceramics. Biomaterials.

[43] Thomson TS, Berry JE, Somerman MJ, Kirkwood KL (2003). Cementoblasts maintain expression of osteocalcin in the presence of mineral trioxide aggregate. J Endod.

[44] Wu B-C, Youn S-C, Kao C-T, Huang S-C, Hung C Jr, Chou M-Y, Huang T-H, Shie M-Y (2015). The effects of calcium silicate cement/fibroblast growth factor-2 composite on osteogenesis accelerator in human dental pulp cells. Journal of Dental Sciences.

[45] Asgary S, Eghbal MJ, Ehsani S (2010). Periradicular regeneration after endodontic surgery with calcium-enriched mixture cement in dogs. J Endod.

[46] Asgary S, Eghbal MJ, Parirokh M, Ghanavati F, Rahimi H (2008). A comparative study of histologic response to different pulp capping materials and a novel endodontic cement. Oral Surg Oral Med Oral Pathol Oral Radiol Endod.

[47] Mente J, Leo M, Panagidis D, Ohle M, Schneider S, Lorenzo Bermejo J, Pfefferle T (2013). Treatment outcome of mineral trioxide aggregate in open apex teeth. J Endod.

[48] Rahimi S, Mokhtari H, Shahi S, Kazemi A, Asgary S, Eghbal MJ, Mesgariabbasi M, Mohajeri D (2012). Osseous reaction to implantation of two endodontic cements: Mineral trioxide aggregate (MTA) and calcium enriched mixture (CEM). Med Oral Patol Oral Cir Bucal.

[49] Samiee M, Eghbal MJ, Parirokh M, Abbas FM, Asgary S (2010). Repair of furcal perforation using a new endodontic cement. Clin Oral Investig.

[50] Asgary S, Eghbal MJ (2013). Treatment outcomes of pulpotomy in permanent molars with irreversible pulpitis using biomaterials: a multi-center randomized controlled trial. Acta Odontol Scand.

[51] Malekafzali B, Shekarchi F, Asgary S (2011). Treatment outcomes of pulpotomy in primary molars using two endodontic biomaterials A 2-year randomised clinical trial. Eur J Paediatr Dent.

[52] Jaberiansari Z, Naderi S, Tabatabaei FS (2014). Cytotoxic effects of various mineral trioxide aggregate formulations, calcium-enriched mixture and a new cement on human pulp stem cells. Iran Endod J.

[53] Küçükkaya S, Görduysus MO, Zeybek ND, Müftüoğlu SF (2016). In Vitro Cytotoxicity of Calcium Silicate-Based Endodontic Cement as Root-End Filling Materials. Scientifica.

[54] Mozayeni MA, Milani AS, Marvasti LA, Asgary S (2012). Cytotoxicity of calcium enriched mixture cement compared with mineral trioxide aggregate and intermediate restorative material. Aust Endod J.

[55] Saberi EA, Karkehabadi H, Mollashahi NF (2016). Cytotoxicity of Various Endodontic Materials on Stem Cells of Human Apical Papilla. Iran Endod J.

[56] Keiser K, Johnson CC, Tipton DA (2000). Cytotoxicity of mineral trioxide aggregate using human periodontal ligament fibroblasts. J Endod.

[57] Vajrabhaya LO, Korsuwannawong S, Jantarat J, Korre S (2006). Biocompatibility of furcal perforation repair material using cell culture technique: Ketac Molar versus ProRoot MTA. Oral Surg Oral Med Oral Pathol Oral Radiol Endod.

[58] Yuan Z, Peng B, Jiang H, Bian Z, Yan P (2010). Effect of bioaggregate on mineral-associated gene expression in osteoblast cells. J Endod.

[59] Yan P, Yuan Z, Jiang H, Peng B, Bian Z (2010). Effect of bioaggregate on differentiation of human periodontal ligament fibroblasts. Int Endod J.

[60] De-Deus G, Canabarro A, Alves G, Linhares A, Senne MI, Granjeiro JM (2009). Optimal cytocompatibility of a bioceramic nanoparticulate cement in primary human mesenchymal cells. J Endod.

